# EvoMol: a flexible and interpretable evolutionary algorithm for unbiased de novo molecular generation

**DOI:** 10.1186/s13321-020-00458-z

**Published:** 2020-09-16

**Authors:** Jules Leguy, Thomas Cauchy, Marta Glavatskikh, Béatrice Duval, Benoit Da Mota

**Affiliations:** 1 Laboratoire LERIA, UNIV Angers, SFR MathSTIC, 2 Bd Lavoisier, 49045 Angers, France; 2 Laboratoire MOLTECH-Anjou, UMR CNRS 6200, UNIV Angers, SFR MATRIX, 2 Bd Lavoisier, 49045 Angers, France

**Keywords:** Chemical space exploration, Organic molecular materials

## Abstract

The objective of this work is to design a molecular generator capable of exploring known as well as unfamiliar areas of the chemical space. Our method must be flexible to adapt to very different problems. Therefore, it has to be able to work with or without the influence of prior data and knowledge. Moreover, regardless of the success, it should be as interpretable as possible to allow for diagnosis and improvement. We propose here a new open source generation method using an evolutionary algorithm to sequentially build molecular graphs. It is independent of starting data and can generate totally unseen compounds. To be able to search a large part of the chemical space, we define an original set of 7 generic mutations close to the atomic level. Our method achieves excellent performances and even records on the QED, penalised logP, SAscore, CLscore as well as the set of goal-directed functions defined in GuacaMol. To demonstrate its flexibility, we tackle a very different objective issued from the organic molecular materials domain. We show that EvoMol can generate sets of optimised molecules having high energy HOMO or low energy LUMO, starting only from methane. We can also set constraints on a synthesizability score and structural features. Finally, the interpretability of EvoMol allows for the visualisation of its exploration process as a chemically relevant tree. 
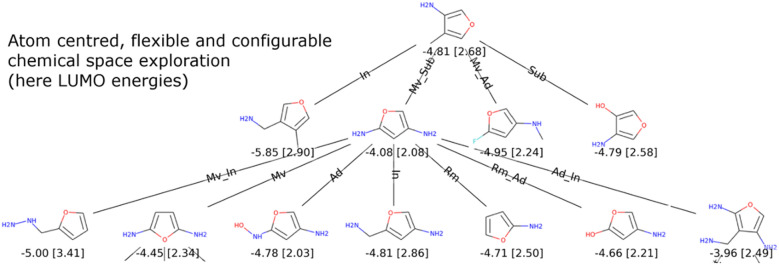

## Introduction

One of the main objectives of chemical research is to find a molecule that has desired properties for a given application. However, the molecular space being immeasurable, one needs to define strategies to efficiently explore its relevant parts. Even an incomplete enumeration of the chemical space limited to 17 heavy atoms (C, N, O, S and halogens) already leads to more than 160 billion compounds [1]. To tackle this problem, we will see that many methods have been proposed, adapting recent advances in deep learning and in reinforcement learning, or using more classical optimisation methods such as evolutionary algorithms. Actually, fully automated de novo molecular generation is a subject that has regained considerable attention [2, 3].

In the 1990–2000s, evolutionary algorithms were already used for de novo molecular generation [4]. To limit the number of steps and to improve the likeliness of the solutions, they were commonly based on the combination of fragments rather than mutating the molecules at atomic level. The interest in evolutionary algorithms has decreased with the emergence of deep learning for molecular generation, although very recently, a new and efficient fragment based method was designed [5]. In the mid 2010s, Aspuru-Guzik and coll. proposed two deep learning architectures, a Generative Adversarial Network (GAN) and a Variational Auto Encoder (VAE) [6, 7]. After the deep learning successes in image generation, one could have hoped for a revolution in the molecular generation [8].

In fact, several deep learning architectures were then published for de novo molecular generation. Among them, autoencoders are trained to convert points of their latent space to SMILES [7, 9–11] or molecular graphs [12, 13]. Their latent space can then be explored to provide new solutions. Recently, Yuan * et al.* proposed a transfer learning approach to take advantage of the abundant data in pharmaceutical-type molecules and generate chemically feasible solutions for materials [14]. Other neural networks are designed to build sequentially molecular graphs [15] or molecular Cartesian coordinates [16]. We should mention here that Bayesian methods have also been proposed successfully even for organic materials to generate small gap molecules [17]. All these methods highly depend on training data. This causes an issue in terms of exploration ability, since the accessible chemical space is implicitly biased by the data.

Authors have therefore suggested to use reinforcement learning to push those boundaries and orient the search towards desired properties. GAN can be associated with reinforcement learning to optimise properties, while using their discriminator to evaluate the likeliness of the solutions [6, 18]. This setting provides credible molecules in the neighbourhood of the training dataset. Recurrent neural networks can also be tuned by REINFORCE [19] or Monte-Carlo Tree Search [20, 21] algorithms to optimise properties. Kwon et al. proposed recently a reinforced VAE for the generation of molecular graphs [22]. Some authors also take advantage of the sequential nature of deep reinforcement learning to filter invalid solutions at each step and thus guarantee the validity of the generated molecules [23–25]. These methods have been able to achieve higher performances by using increasingly complex architectures, which has led to a decrease in their interpretability. Furthermore, only the work of Zhou et al. is independent of a starting dataset for training [24].

There is renewed interest with recent evolutionary methods leading to competitive results [26–28]. This interest is motivated by their simplicity as well as their higher level of interpretability. In evolutionary algorithms, the chemical subspace to be explored can be defined through the choice of the domains and operators. These rules can be set by different approaches, such as a grammar [26, 28] or a statistical definition of mutations [27]. Recent evolutionary algorithms tend to use mutations closer to the atomic level, as it allows a smoother exploration of the chemical space. The implementation of such methods was made possible by the emergence of tools such as RDKit, simplifying the programming and allowing for easy sanity testing of the molecules [29].

The objective of our work is to design a molecular generator capable of exploring known as well as unfamiliar areas of the chemical space. Between drug-like generation and organic materials, the chemical space of interest is different [30]. The molecular materials space is less known and has probably been only intensively searched around the few known successes. Thus, our method must be flexible to adapt to very different problems. Furthermore, we have previously found that quantum mechanics datasets for small organic molecules (QM9 and PC9) present generalizability issues [31]. There is currently no reliable and diverse training dataset for organic materials. Therefore, our method has to be able to work with or without the influence of prior data and knowledge. Moreover, the exploration should be as interpretable as possible to allow for structure property studies and for chemical interpretation of the building process. Evolutionary algorithms are fitted to tackle this problem, as they are independent of starting data and as the definition of their search space is explicit.

In this paper, we present EvoMol, a new generic and simple molecular generation method using an evolutionary algorithm to sequentially build molecular graphs. To be able to search a large part of the chemical space, we define an original set of 7 local and chemically meaningful mutations.

We calibrate our methodology using several properties of the literature that are classic and fast to compute. In this way we can also compare ourselves with state of the art methods. Targets are the QED [32], penalised logP [33], SAscore [34] and CLscore [35]. They are functions that encompass physicochemical properties and structural features to roughly estimate the drug-likeness and synthetic accessibility. We show that our method is able to optimise these properties to high scores. EvoMol even outperforms state-of-the-art methods on penalised logP optimisation. As a more complete benchmark, we use the set of goal-directed functions defined in GuacaMol [36], on which EvoMol provides very competitive results.

To demonstrate the flexibility of EvoMol, we tackle a very different objective issued from the organic molecular materials domain. Usually in this domain, the aim is to find a molecule with given electronic properties. It can be for example a desired HOMO or LUMO level or an UV-visible absorption / emission range. Such properties require quantum chemistry calculations to be precisely assessed. The objective function cost is therefore huge and increases rapidly with the molecular size.

We show that our method achieves excellent performances on the optimisation of these electronic properties. We demonstrate that starting from methane, which has one of the lowest HOMO and highest LUMO in energies, our algorithm can generate sets of optimised molecules i.e. having high HOMO or low LUMO energies. We can also set constraints on a synthesizability score and structural features. Finally, we propose a chemically relevant visualisation tree to follow the exploration process.

## Methods

### Graph representation

Beyond the question of the method, the representation of the solutions plays a crucial role for molecular properties optimisation. Ideally, the representation should allow for the definition of a rich, interpretable and valid neighbourhood while requiring small computational cost. In practice, such molecular representation does not exist, and a trade-off must be found.

Early methods mainly represent the molecules as SMILES [37], a linear text representation allowing easy processing with sequential methods using recurrent neural networks and reinforcement learning [19, 20]. Solutions can also be directly derived from the grammar of SMILES [26]. However, methods building SMILES character by character cannot filter invalid solutions in intermediate steps. The reason is that they need to explore the space of invalid solutions to perform ring closure and branching (bonding an atom with more than two other atoms). As a result, these methods do not guarantee the validity of their solutions. Another text representation named SELFIES was recently proposed as an alternative offering a validity guarantee, at the cost of an increased complexity [38]. It was successfully used for molecular generation [28].

The other common representation is the molecular graph. It can be extracted from the latent space of deep learning methods [12, 13, 22]. It can also be built sequentially, using reinforcement learning [15, 23–25] or using evolutionary algorithms [27]. Working sequentially allows for a strict control on the validity of the solutions. By filtering invalid actions at every step, the validity of the molecules can be guaranteed for each intermediate and final step. Furthermore, the sequential approach makes it possible to define a chemically meaningful neighbourhood of molecules which enhances the interpretability of the exploration. That is why we define our method using a molecular graph representation. It is worth noting here that our graphs consider hydrogens implicitly. It means that atoms are bonded with hydrogens until the defined valency is reached.

### Algorithm

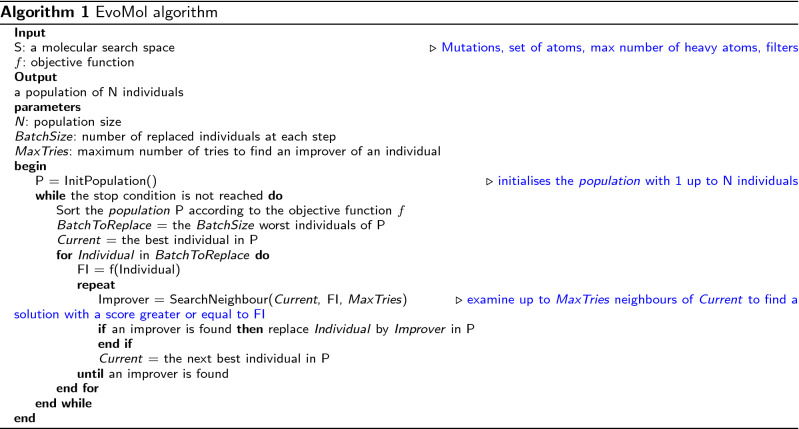



The behaviour of EvoMol is described in Algorithm 1. At first, the chemical subspace to explore is defined through the choice of the mutations on the molecular graph, the set of atoms, the molecular size limit and the filter rules. Then, the population is initialised with one or more molecules up to the maximum population size. The choice of the initial population is an often underestimated but crucial element of the knowledge available to the model. It can have a huge impact on the final performance of the model. The knowledge introduced in the form of an initial population can compensate ill-defined objective functions for very specific and complex tasks (as we will see in "Case 2: GuacaMol" section).

At this point, the main task of molecular generation starts. The population is sorted according to the objective function in order to select a batch of the molecules with the worst scores and to identify the best molecules that will be mutated. Several individuals are mutated at each step in order to maintain some diversity. Furthermore, duplicate molecules cannot be inserted in the population, so that the uniqueness of solutions is guaranteed. The equality test is performed by comparing canonical aromatic SMILES with RDKit [29].

To keep the method as simple as possible, we choose to mutate the best-scoring individuals and to replace the worst-scoring. We also adopt a first improver policy, meaning that we impose the mutated individuals to have a better score than the individuals they replace, although we also insert individuals with same score to allow for plateau exploration. We only search for an improver a maximum fixed number of times. If no improver is found, we start again the search on the next individual to be mutated, until the expected number of individuals are replaced during the step, or no individual is left to be mutated.

We define the mutation operator as a successive use of randomly selected actions on the molecular graph (see "Actions on molecular graphs" section) in order to accelerate the exploration and to give the method good ability to escape from local extrema. The random selection is made by drawing the action type first, then by drawing the actual action, both with uniform laws to avoid assumptions on the chemical space. The number of successive actions is randomly drawn between 1 and a parameter defining the maximum value. As the optimisation is only guided by the objective function, our method is easily generalisable to multiple molecular optimisation problems.

EvoMol is able to start the optimisation with an initial population containing less individuals than the maximum allowed size. In this case, mutated individuals are simply added during first steps until the expected population size is reached. The number of added individuals during each step of this process is bounded by the number of available individuals to be mutated. We use this process to perform the optimisation of molecular properties starting solely from the methane molecule, so that it is not biased by prior knowledge in the form of an initial population. Virshup et al. had already shown before, that starting from benzene and cyclohexane does not hinder the exploration of the chemical space [39].

### Actions on molecular graphs

**Fig. 1:**
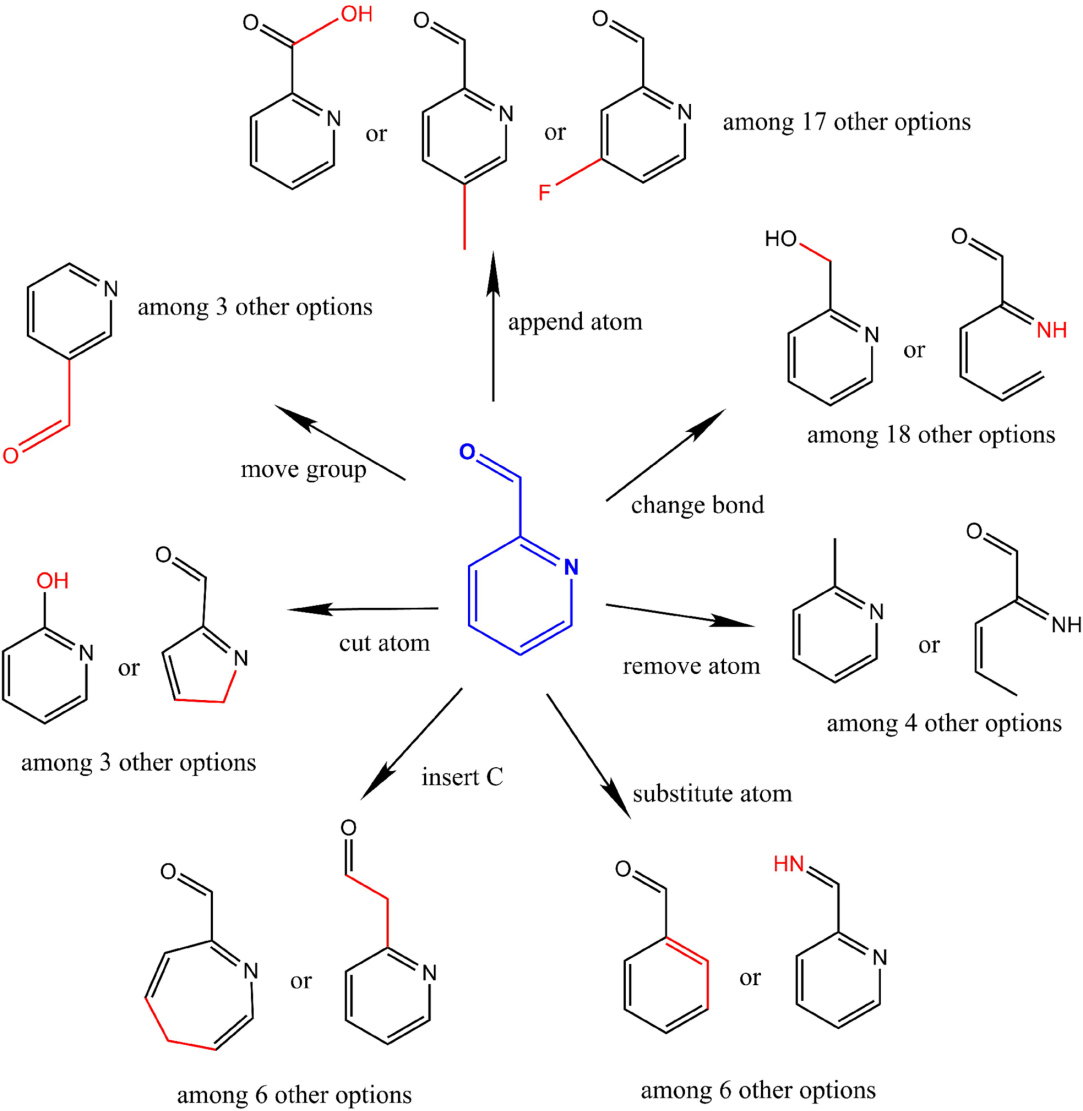
Application of the primary (append atom, change bond, remove atom) and secondary actions on the molecular graph of the 2-Formylpyridine, using C, N, O or F atoms

Among the actions on molecular graphs that we define and present in Fig. 1, three actions have a primary effect on the molecules. *Append atom* adds an atom with a single bond to an existing atom of the graph. *Remove atom* removes an atom from the graph. *Change bond* changes the type of a bond. The possible bond types are none (no bond), single, double and triple. With these three actions, it is possible to reach a large portion of the molecular space. However, it require multiple steps to perform changes which could be considered as chemically rudimentary.

We therefore define a set of secondary actions, using sequences of primary actions to create small shortcuts in the chemical space in order to accelerate the exploration. It should be noted that these actions do not extend the accessible chemical space. *Substitute atom type* changes the type of an atom of the graph. *Insert carbon* corresponds to the insertion of a carbon atom between two existing atoms sharing a bond. The initial bond between the two atoms is removed and the carbon atom is linked to them with two single bonds. Only carbon can be inserted, as it is the backbone of organic chemistry and in order to limit the action space size. *Cut atom* removes an atom sharing exactly two bonds with two atoms not sharing a bond with each other. The two remaining atoms are then linked with a single bond. Finally, *Move group* relocates a functional group to a different atom of the molecular graph, while conserving the type of the removed bond. Functional groups are subgraphs only linked to the rest of the molecule by a single edge (a chemical bond). Fig. 1 illustrates examples of these actions on the 2-Formylpyridine molecule. Note that our actions are both constructive and destructive so that the research can be lead from any starting point.

The successive application of our defined actions can be followed in an exploration tree. We will see that this approach allows relevant and interpretable figures. So, we ruled out recombination operations between solutions, which would result in less readable visualisations. We do not design any specific action to handle aromaticity, which is considered implicitly through the Kekulé forms that can be created. To guarantee the validity of the generated molecules, we define two *a priori* filters discarding actions leading to invalid molecular graphs, without a need to apply the actions to assess the validity. The first filter prevents the dislocation of the molecular graph by prohibiting the use of actions leading to the formation of additional connected components. The second filter prevents the use of actions leading to incorrect valences. Since hydrogens are considered implicitly, only hypervalence leads to inconsistency. However, the implicitness of hydrogens complexifies the processing of charged atoms with our method. We do not define any action leading to their creation nor neutralisation, but we accept ions and zwitterions as input graphs by filtering the actions having an effect on the bonds of charged atoms, except for atom removal. Therefore, ionic subgraphs can be conserved or removed while actions are modifying the molecular graph.

For some applications, it can be desired to mutate a molecule while freezing a set of its atoms. Our method can be easily adapted to this problem by defining a third filter with the following effect. *Substitute atom type* and *cut atom* can only be applied on a mutable atom. *Change bond*, *insert carbon* and *move group* can only be applied on a couple of atoms containing at least one mutable atom. This approach is illustrated by an example where we consider an optimisation of HOMO and LUMO energy levels for furane derivatives (see "Case 3: HOMO/LUMO optimisation" section).

### Experiments

As stated in the introduction, we assess our method using several properties commonly used as objective functions. QED is a metric evaluating the drug-likeness based on the similarity of the distributions of a set of properties with known drugs [32]. SAscore is defined as an estimation of synthetic accessibility based on the similarity with structural features observed in a subset of the PubChem [40] and penalising uncommon rings and numerous stereo centres [34]. It is usually ranged between 1 and 10. For comparison with previous methods, we optimise a normalised version noted SAscore^‡^ ranging between 0 and 1. For both versions, 1 is the best possible score. Penalised logP (plogP) corresponds to the octanol-water partition coefficient penalised by the SAscore and the presence of a large ring [33]. In the original article, these properties are normalised on a subset of the ZINC dataset [41], but it has also been optimised without normalisation [24]. Likewise, we note plogP^‡^ the normalised version. We have adapted the implementations of You et al. and Zhou et al. respectively [23, 24]. We also use our method to optimise the recently proposed CLscore [35]. It is another drug-likeness evaluation metric, based purely on the structural similarity with a subset of biologically active molecules in the ChEMBL [42]. To define the CLscore, weights are assigned on molecular subgraphs, named shingles, proportionally to their representation in the chosen subset of ChEMBL. The score of a molecule is computed by performing the mean of the weights of its shingles. The shingles take into account the circular substructures but contrary to the SAscore, the CLscore does not penalise explicitly uncommon rings and numerous stereo centres. CLscore is computed adapting the Bühlmann et al. implementation. All properties use RDKit for their computation [29].

The previous metrics, especially plogP and QED, have been widely used to compare the molecular generation methods. However, they are optimised using different search space definitions in terms of available atom types and maximum sizes of molecules. This can have a huge effect on the results, particularly for plogP. In this article, we only compare EvoMol with methods using a similar space search definition.

Because the optimisation of the previous properties can be trivial and in order to homogenise the evaluation of the methods, a benchmark named GuacaMol has been recently proposed [36]. We evaluate our method with its goal-directed benchmark, composed of 20 various maximisation tasks (albeit mainly drug-generation oriented). These tasks have several generation purposes, including the rediscovery of known drugs, molecules similar to a specific target or all isomers of given formulas. Some tasks combine similarity and property objectives and some add structural constraints. All similarities are computed using molecular fingerprints.

As a proof of concept in the domain of organic molecular materials, we also use our method to optimise two electronic properties, the HOMO and LUMO energy levels. Contrary to the previously presented benchmarks that depends on fast evaluations of fitness functions, HOMO and LUMO energies depend on quantum mechanics calculations with a heavy computational cost. Here, geometric optimisations are carried out with the Gaussian09 program and default parameters [43]. The B3LYP hybrid functional and the small basis set 3-21G are chosen to reduce the computational cost [44]. We only perform DFT optimisation on molecules conserving the same SMILES after molecular mechanics 3-D coordinates generation using Open Babel [45]. Likewise, we consider a DFT result as valid only if the SMILES remained identical after the geometric optimisation.

For all these objective functions, different initial conditions and parameters define our algorithm. We test the impact of the population size, its initial state, the atomic types set and the actions set. For QED, plogP, SAscore and CLscore populations sizes ranging from 1 to 457k are tested. For GuacaMol and electronic properties, we use either a relevant starting population (a ChEMBL subset or a furane core) or a simple methane molecule to assess the initial knowledge impact and to perform a demonstration in challenging conditions.

## Results and discussion

### Case 1: QED, pLogP, SAscore, CLscore

The first case consists in optimising the set of fast metrics containing QED, both plogP versions, SAscore^‡^ and CLscore. To provide a baseline, we run our algorithm to maximise each of these properties with a population size of 1, starting with the methane. This corresponds to a simple hill-climber algorithm with a first improver policy, as a unique solution is mutated until an improver is found. It means that a direct pathway must be found in the molecular space between the methane starting point and a molecule solution.

We conduct our space exploration on molecules containing C, N, O, F, P, S, Cl or Br atoms. For a fair comparison with other methods, we limit the sizes of the molecules to 38 heavy atoms. As the plogP is sensitive to the set of atoms, we perform a second run for its optimisation using only C, N, O or F atoms. The mutation is defined as the application of up to 2 successive actions, and the algorithm is ran for 1500 steps, with up to 50 tries to find an improver at each mutation. All experiments are ran 10 times and results are averaged.

The results of the single individual optimisation (see Table 1) show that this setting outperforms state-of-the-art methods with comparable conditions on the plogP optimisation, even using only C, N, O and F atoms. For QED, very good values are obtained. We also evaluate our method with a better exploration ability, by evolving a population of 1000 individuals on the same benchmark, using the same parameters except for the number of replaced individuals per step which is raised to 10. The population is initialised with a single methane molecule.

**Table 1:** Best scores for classical objectives

Method		QED	plogP	plogP^‡^
ChemGE [26]				5.88
GB-GA [27]				7.40
GCPN [23]		0.948		7.98
MolDQN [24]		0.948	11.84	
Zhang et al. [25]		0.954	12.96	
EvoMol	pop. 1	0.922	14.49	11.19
	pop. 1000	0.948	18.06	13.79
EvoMol {CNOF}	pop. 1	0.902	13.88	11.19
	pop. 1000	0.948	13.88	11.19

The values for state-of-the-art methods are reported from original articles. The results for EvoMol are the average of the maximum over 10 executions. ^‡^ The Symbol stands for normalised version of plogP

The mean scores for SAscore and QED of all 1000 generated molecules are reported in Table 2. All scores are optimised to a high mean value, generally better than the literature. For all these objectives, our method is able to nicely generate optimised sets and by construction, all the solutions are different. It is clearly an improvement upon published methods. Besides, optimising a population of 1000 individuals generally improves the top scores (see Table 1).

**Table 2:** Mean scores and proportion of unique solutions for QED and the normalised version of the SAscore

Method	QED		SAscore	
	Mean	Unique %	Mean	Unique %
ORGAN [6]	0.52	69.4	0.83	45.9
MOLGAN [18]	0.62	2.2	0.95	2.1
EvoMol	0.948	100.0	0.95	100.0

The values for state-of-the-art methods are reported from [18]. Results for EvoMol are the average of the mean score over 10 executions

Beyond our ability to optimise an objective, it can be interesting to look at the efficiency in time or in the number of calls to the evaluation function of our method. Evolutionary algorithms have already demonstrated an ability to obtain good plogP values in 30 seconds when deep generative methods need several hours [27]. EvoMol is able to find in 30 seconds a value of 10.34 ($\pm 0.43$) in plogP on average over 10 runs with a population of a 1000. A score of 11.19 ($\pm 0.00$) is obtained on a single molecule with a limit to 1000 for the number of calls to the evaluation function starting from the methane. In average only 400 evaluations are needed for this problem.

**Fig. 2:**
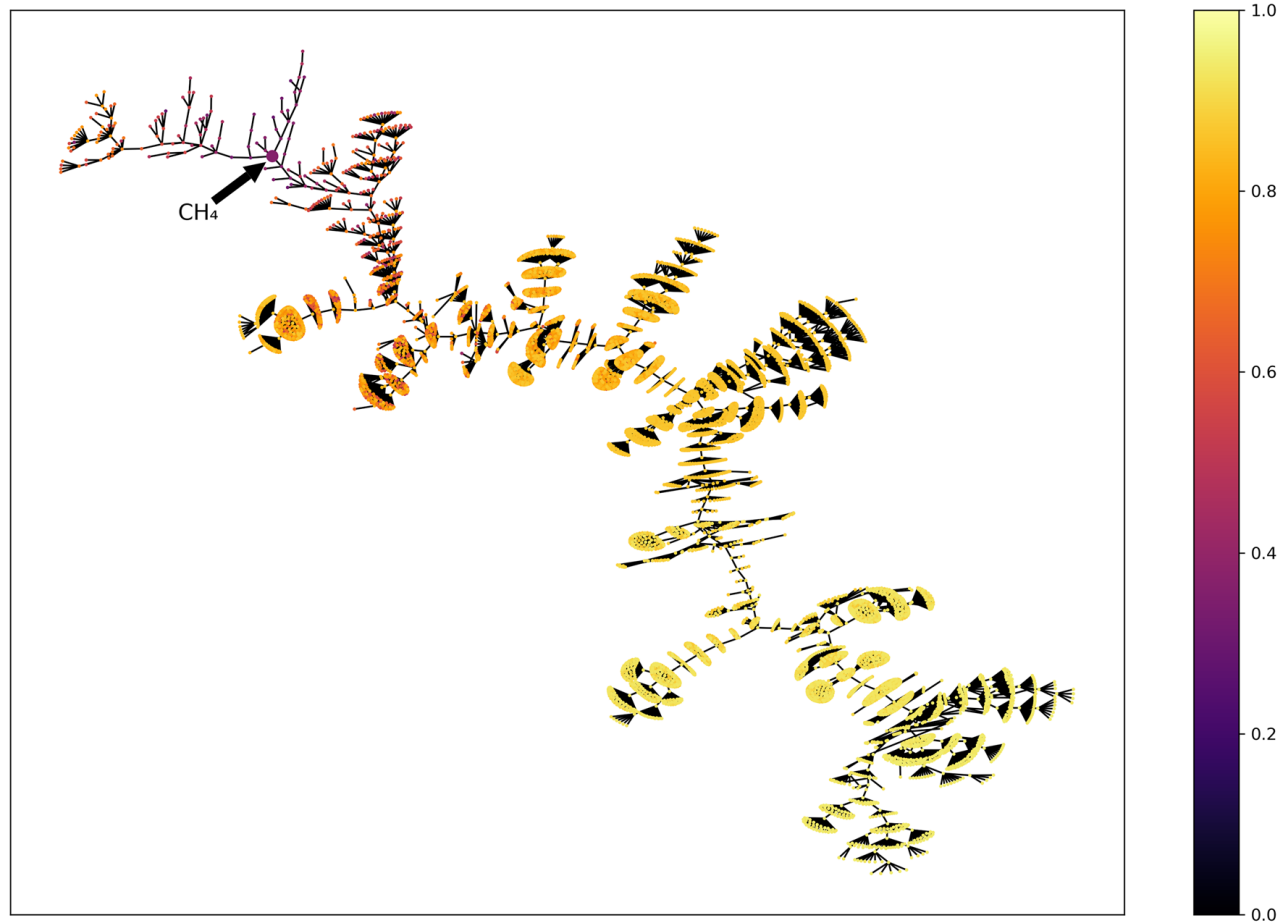
Exploration tree of a QED optimisation run after 700 steps. The starting point (methane) is represented as a large dot indicated with the arrow. Edges represent mutations that lead to an improvement in the population. Solutions are coloured according to their score

The evolutionary approach on molecular graphs is an efficient method to find needles in a haystack [46]. Thanks to our sequential and atom centred process, we can also visualise the progression of the exploration, allowing a better interpretation of the results (see Fig. 2). It shows the ability of our method to intensify over promising areas of the chemical space and to ignore others. In particular, it can be observed that the exploration of some areas close to the starting point was quickly abandoned, whereas the space of high-scoring solutions was intensively searched.

**Fig. 3:**
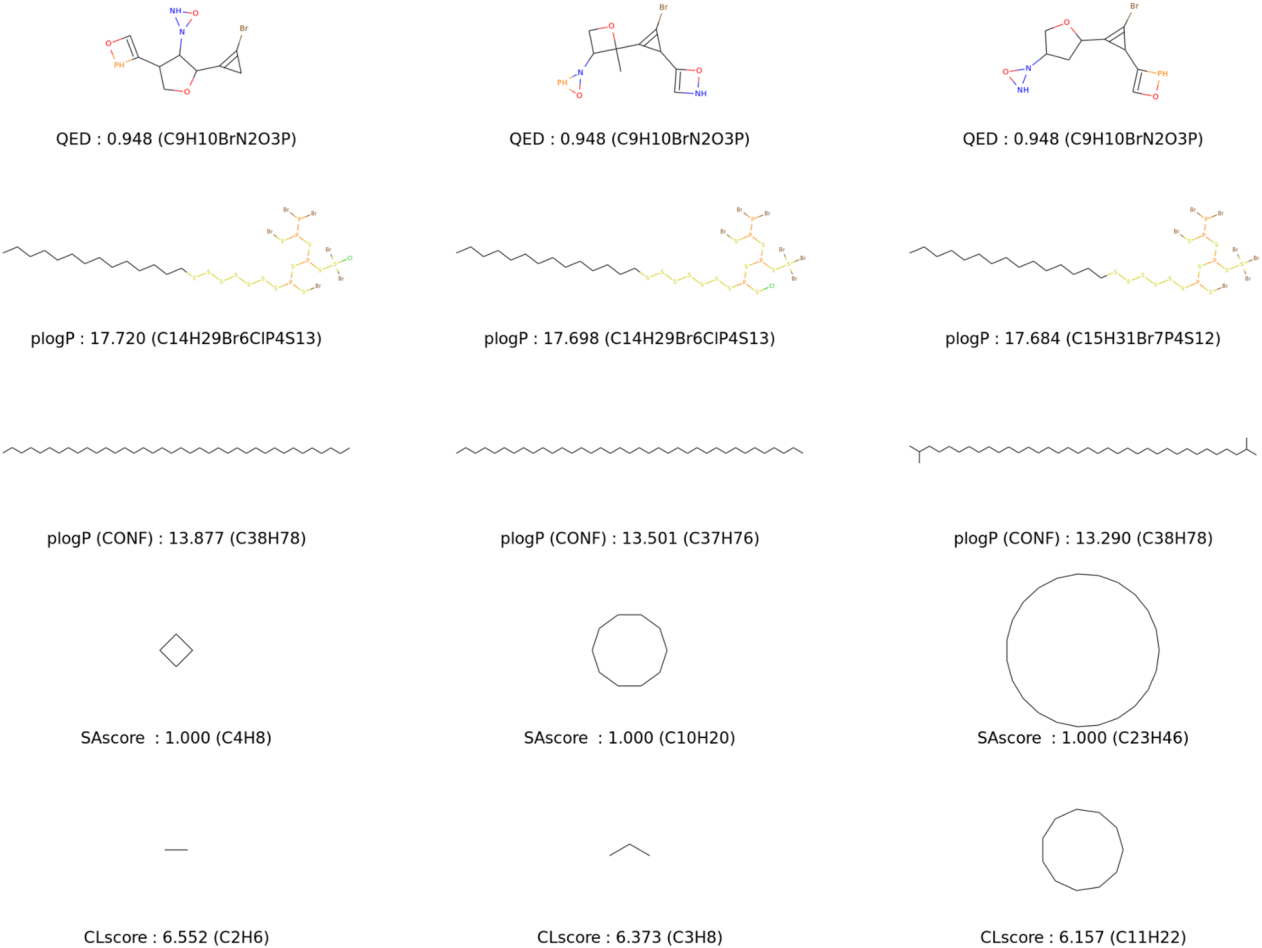
The three best scoring molecules found while optimising a set of simple properties using a population size of 1000. Shown examples are drawn randomly if the best score is held by several molecules. They are sampled from the final population of a randomly selected execution among the 10 performed runs for each property. plogP^‡^ examples are not shown as they are very similar to the plogP examples

The same observation can be made from Fig. 3, which represents the top scoring molecules found for each property. It shows that the best solutions are quite similar to each other. Regarding specific properties, it appears that the QED leads to quite unrealistic molecules in terms of drug-likeness, containing many different hetero-atoms. QED is a multi-objective function that favours compounds with little bits of drug-likeness [32]. It rewards some hydrogen bonds donors and acceptors, some cycles and a medium logP. Therefore, we found chimeras that present a little bit of “everything”.

More interestingly, we can analyse more closely the solution for the plogP problem since it is a straightforward function. A maximised logP represents a soluble compound in octanol. The highest atomic contribution proposed by Wildman et al. [47] and used by RDKit, are iodine (0.886), phosphorous (0.861), bromine (0.846), chlorine (0.690) and aliphatic sulfur (0.648). Furthermore, taking into account the number of heavy atoms favours long alkyl chains to profit from the positive effects of the hydrogens. Therefore, the best scaffold will be based on primary and secondary aliphatic carbons (0.144 each). The penalised version includes the SAscore but the good score of the starting alkyl chain compensates the penalty received with the sulfur, phosphorous and bromine part. That is why we found long alkyl chains with H, C, N, O and F and some kind of complex surfactants when Br, Cl, S and P are also considered.

**Fig. 4:**
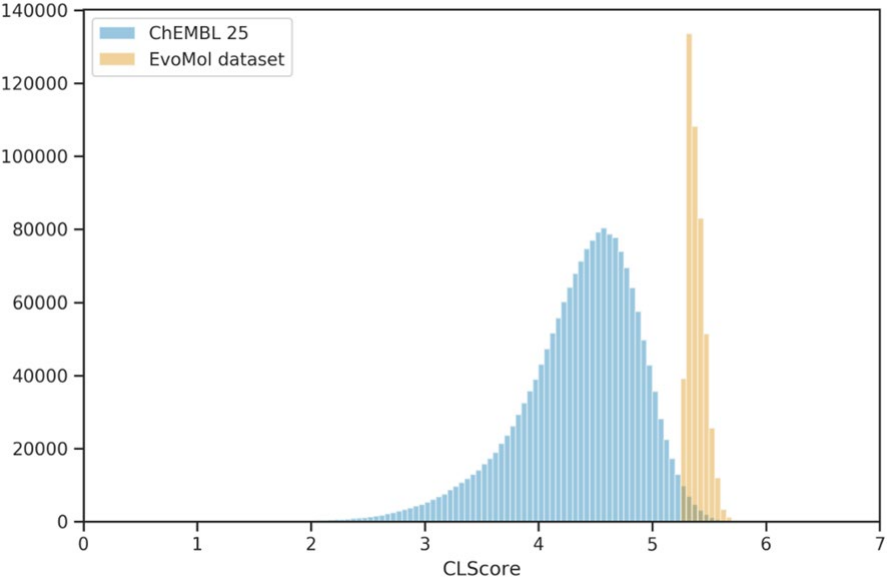
Distribution of the CLscore in ChEMBL 25 (blue) and in the dataset generated with EvoMol containing 457,139 molecules (orange)

We have also tested our ability to generate large sets of molecules with greater chemical feasibility. For this we have chosen the recently proposed CLscore [35]. The CLscore is defined as a structural similarity measure with a subset of 457,139 ChEMBL compounds. Therefore, we impose a population of this size (457,139) with a CLscore maximisation objective, starting solely from methane. The number of replaced individuals per step is set to 4572, *i.e.* 1% of the population size. By this way we obtain a top score of 6.641 and a very high mean of 5.260. As a reference, Bühlmann *et al.* consider a CLscore value $\ge 3.3$ as relevant, since the ChEMBL subset on which the score is defined has its peak at 3.9 [35]. Note that the CLscore distribution on all 1.9 millions small molecules of the ChEMBL 25 present a peak around 4.5 when our 457k solutions correspond to the upper part of this distribution (see Fig. 4).

It appears that EvoMol was able to generate a large set of compounds that are more ChEMBL-like than ChEMBL. Jokes aside, our method has clearly intensified the right part of the distribution and could provide a clever way to generate millions ChEMBL-like solutions. It might also be more relevant to optimise the CLscore to a value between 3 to 5, as it corresponds more or less to the peak in its distribution. Please note, it can be easily achieved with an alkane with few exotic chemical environments. All generated SMILES are available in Additional file 1.

With respect to the synthesizability functions, EvoMol achieves its goal. The optimised SAscore and CLscore compounds, represented in Fig. 3, correspond to either cycloalkanes or small alkanes. Indeed, the alkyl chemical environment is very common in both PubChem and ChEMBL. Since the SAscore and CLscore reward such fingerprints or shingles in the molecule, our method obtains very high scores with such solutions. Both metrics can be used to assess a synthetic accessibility. In a way, this experiment is a success as it converges to almost actual raw materials.

With all these simple metrics, we demonstrate the efficiency of the optimisation performance of EvoMol. However, in chemistry most of the proposed objective functions like the logP are more relevant when optimised to a given range based on statistics [22, 32, 36]. But in this case, the problem becomes even more under-constrained and corresponds to tons of solutions.

The overall interest of these solutions can be considered as weak, as they are either well known or unrealistic. Though it could be tempting to blame a method for such results, we believe the main issue rather lies in the evaluation metrics. Actually, more satisfying results could probably be obtained by tweaking the exploration process so that it leads to a desired subset of the chemical space. However, the fact that high scoring solutions are disappointing raises questions about the metric itself, especially when used as an objective function to be maximised. The GuacaMol benchmark, tested in the following part, defines most of its objective functions as a combination of properties optimised to specific values.

### Case 2: GuacaMol

The second case consists in optimising the goal-directed benchmarks proposed by GuacaMol [36]. Its 20 optimisation tasks consist in, for 6 of them to find identical or similar molecules to known drugs, for 2 of them to generate isomers, and for the other 12 to find solutions satisfying multiple objectives. The tasks are defined in order to provide a various and broad benchmark. Even if this benchmark is more a drug-design approach, it is for now the best comparison tool for molecular generation.

Graph GA, originally designed by Jensen [27] is the genetic algorithm evaluated in the GuacaMol article. We apply the same methodology, using as initial population for each task the 100 best scoring solutions from the filtered dataset provided by GuacaMol. This dataset excludes the molecules that are too close to the targets. To evaluate the influence of the domain specific knowledge introduced with the initial population, we also perform a run with methane as starting point. Finally, we perform another run using only primary molecular graph actions (append atom, remove atom and change bond, see Fig. 1) to study the relevance of the secondary actions. For all GuacaMol experiments, we raise the maximum size of solutions in number of heavy atoms to 50. The other parameters are the same as in case 1, except for the experiments starting from methane and using only primary actions, which are allowed up to 3 actions at each mutation to give them better ability to escape from local extrema. We also raise their steps limit to 3000, since they need more processing to lead to the same state. As before, we run all experiments 10 times, to study their variability.

**Table 3:** Results on the GuacaMol benchmark

Benchmark	SOTA methods				EvoMol				
	SMILES LSTM	Graph GA	CReM [5]	MSO [9]	Primary actions	All actions	From Methane	Best run	*Best* *scores*
Celecoxib rediscovery	*1.000*	*1.000*	*1.000*	*1.000*	0.714	0.978	0.923	*1.000*	*1.000*
Troglitazone rediscovery	*1.000*	*1.000*	*1.000*	*1.000*	0.936	*1.000*	0.676	*1.000*	*1.000*
Thiotixene rediscovery	*1.000*	*1.000*	*1.000*	*1.000*	0.852	0.876	0.695	*1.000*	*1.000*
Aripiprazole similarity	*1.000*	*1.000*	*1.000*	*1.000*	*1.000*	*1.000*	0.964	*1.000*	*1.000*
Albuterol similarity	*1.000*	*1.000*	*1.000*	*1.000*	*1.000*	*1.000*	0.878	*1.000*	*1.000*
Mestranol similarity	*1.000*	*1.000*	*1.000*	*1.000*	*1.000*	*1.000*	*1.000*	*1.000*	*1.000*
$\hbox {C}_{11}\hbox {H}_{24}$	0.993	0.971	0.966	0.997	*1.000*	*1.000*	*1.000*	*1.000*	*1.000*
$\hbox {C}_9\hbox {H}_{10}\hbox {N}_2\hbox {O}_2\hbox {PF}_2\hbox {Cl}$	0.879	0.982	0.940	*1.000*	*1.000*	0.998	*1.000*	*1.000*	*1.000*
Median molecules 1	0.438	0.406	0.371	0.437	0.446	*0.455*	*0.455*	*0.455*	*0.455*
Median molecules 2	0.422	0.432	*0.434*	0.395	0.411	0.417	0.286	0.417	*0.417*
Osimertinib MPO	0.907	0.953	*0.995*	0.966	0.959	0.955	0.911	0.969	*0.978*
Fexonadine MPO	0.959	0.998	*1.000*	*1.000*	0.966	*1.000*	0.981	*1.000*	*1.000*
Ranolazine MPO	0.855	0.920	*0.969*	0.931	0.943	0.966	0.967	0.957	*1.000*
Perindopril MPO	0.808	0.792	0.815	0.834	0.809	*0.845*	0.789	0.827	*0.884*
Amlodipine MPO	0.894	0.894	*0.902*	0.900	0.874	0.867	0.796	0.869	*0.906*
Sitagliptin MPO	0.545	0.891	0.763	0.868	0.943	0.915	*0.946*	0.926	*0.966*
Zaleplon MPO	0.669	0.754	0.770	0.764	0.791	0.791	0.771	*0.793*	*0.810*
Valsartan SMARTS	0.978	0.990	0.994	0.994	*0.999*	0.998	0.000	0.998	*1.000*
deco hop	0.996	*1.000*	*1.000*	*1.000*	*1.000*	*1.000*	0.607	*1.000*	*1.000*
scaffold hop	0.998	*1.000*	*1.000*	*1.000*	0.989	*1.000*	0.655	*1.000*	*1.000*
total	17.340	17.983	17.919	18.086	17.632	18.060	15.298	*18.210*	*18.415*
total MPO only	5.637	6.202	6.214	6.263	6.286	6.339	6.160	*6.341*	*6.544*

SMILES LSTM and Graph GA values are reported form the GuacaMol article. Values for CReM and MSO methods are reported from their respective articles. Values in the three first *EvoMol* columns correspond to the mean scores on 10 executions for different initial conditions and parameters. The best run column reports the values of the best execution. The best scores corresponds for each task to the best encountered value during all executions independently of the run and thus, the totals in this column are virtual

The results can be found in Table 3. In addition to the GuacaMol baseline models, Graph GA [27] and SMILES LSTM [48], we compare ourselves to two recent methods, namely CReM [5] and MSO [9]. The three first columns for EvoMol correspond to the mean scores obtained on 10 runs for each initial conditions and parameters. The *best run* column contains the values of the run with best total score across all 30 runs. All generated SMILES across all runs are availalbe in Additional file 2. The *best scores* column corresponds to the best score for each single benchmark independently of the run. First, it can be observed that the execution of EvoMol starting from methane has an important liability on some tasks, although it performs very well on others. Especially, it obtains a null score on the Valsartan SMARTS benchmark. This can be easily explained, as this problem is defined as a geometric mean containing a binary constraint on the presence of a complex structure. Therefore, our model with no prior knowledge is not guided to find the structure and remains at the lowest score. A single molecule with this structure as a starting dataset would be sufficient to allow our method to go beyond this minimum. This benchmark illustrates the importance of a well-defined evaluation function, which conditions performance as much as actions.

The 6 rediscovery and similarity tasks are designed to recreate the developed structure of a molecule, even a complex one. We can observe that these objective functions based on ECFC4 or FCFC4 can lead the methane up to or in the neighbourhood of such compounds, albeit not all the time and with difficulty. It is an interesting task if we want to search around a specific three dimension architecture. In our case, it is a very complicated objective since we restrict ourselves to atom-centred operations, when these evaluation functions are based on chemical functions fingerprints. They are therefore not continuous or smooth enough to methodically guide our method. We need too many actions and very specific actions to get out of local optimums.

Compared to the methods of literature, EvoMol has better results on isomers benchmarks ($\hbox {C}_{11}\hbox {H}_{24}$ and $\hbox {C}_9\hbox {H}_{10}\hbox {N}_2\hbox {O}_2\hbox {PF}_2\hbox {Cl}$), for which a set of different solutions satisfying a molecular formula must be found. This shows again its ability to intensify thoroughly over areas of the chemical space, thanks to the local mutations. For the median objectives that are composed of two competing goals, we obtain good results. This can be useful in the optimisation of materials, which is often based on a balance between antagonistic properties.

In the end, the most interesting objectives are the multi-property objectives (MPO). On the one hand, no method seems to find the best solutions on these benchmarks, resulting in high variability between the methods (see Table 3). On the other hand, they correspond to chemically more realistic problems, as they are defined as the optimisation of specific properties in the neighbourhood of target molecules. Actually, they include some similarity. Less constraining than the task of rediscovery, it can represent in the case of molecular organic materials a constraint on a piece of molecule or chemical functions. The MPO include also criteria on physico-chemical properties or composition. Generally there is the logP which is based on atomic contributions depending on the chemical environment. We have seen that this objective is easily achieved. The other property often used is the Topological Polar Surface Area (TPSA) which focuses on the chemical environments of nitrogen and oxygen (RDKit version). There may be some redundancy with the ECFCs4 associated with these atoms in the goal of similarity. Sometimes specific targets have been added such as cycle number, chemical formula, fluorine number.

It can be observed in Table 3 that EvoMol outperforms all methods on these MPO benchmarks. It obtains the best total MPO score of the literature starting from the ChEMBL population and a score in par with state-of-the-art methods starting from methane. Furthermore, the column *best scores* shows that EvoMol can find top scores on 6 of these 7 benchmarks. Evolutionary algorithms are known to be very effective methods to solve combinatorial optimisation problems with contradictory objectives such as the MPO benchmarks. EvoMol is therefore very fitted for these tasks.

**Fig. 5:**
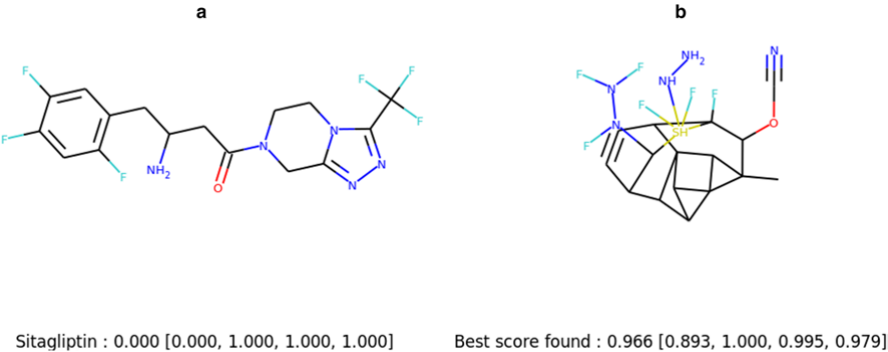
Study of Sitagliptin MPO benchmark. **a** Sitagliptin molecule. **b** Best score found using EvoMol. Scores are reported under the molecules : Sitagliptin MPO score (similarity score, logP score, TPSA score, isomer score)

The Sitagliptin MPO task consists in finding an isomer of Sitagliptin with the same logP and TPSA values but as different as possible in terms of ECFC4 fingerprints. EvoMol succeeds in finding a molecule with by far the best score (see Table 3). The Sitagliptin and our best score are represented in Fig. 5. Our method was able to find radically different chemical environments for the N and O, even adding a sulfur atom, while maximising the logP and TPSA scores. Interestingly our best solutions come from experiments using the methane molecule as a starting point. Not having prior knowledge, allows here to explore more efficiently the remote chemical space of the Sitagliptin.

**Fig. 6:**
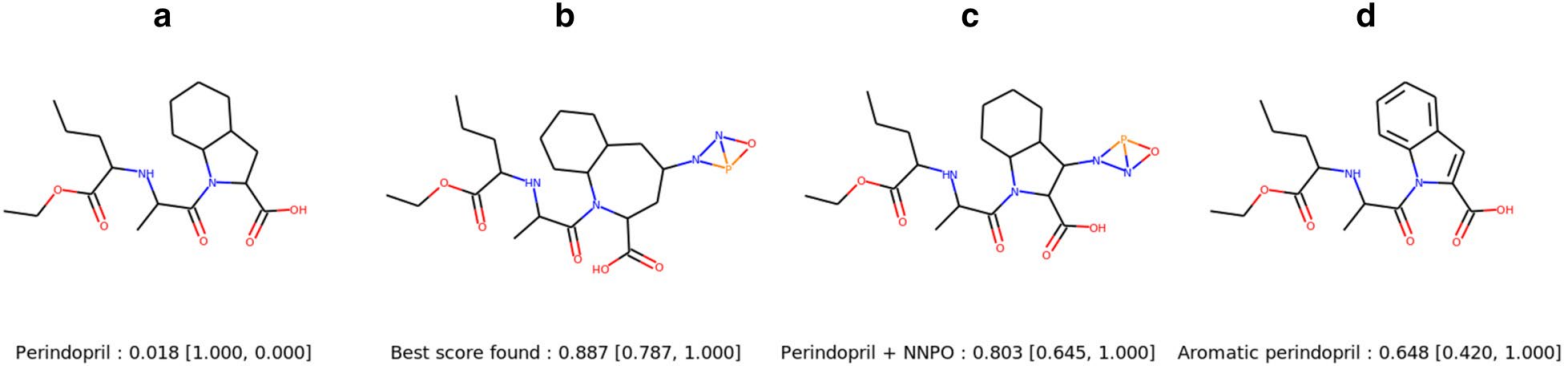
Study of Perindopril MPO benchmark. **a** Perindopril molecule. **b** Best score found using EvoMol. **c**, **d** Hypothetical solutions. Scores are reported under the molecules: Perindopril MPO score (similarity score, # aromatics rings score)

The results for the task Perindopril MPO, which consists in finding a molecule similar to the Perindopril with two aromatic rings, deserves also a thorough analysis. Its structure and our best solution are drawn in Fig. 6. The alkyl part is correctly reproduced whereas the two saturated rings include one extra carbon each. The addition of a peculiar bicycle of O, N and P considered by RDkit as 2 aromatic rings (NNP and NPO) allows for the best score in this sub task. Surprisingly if we consider the real Perindopril molecule with the small aromatic part or with two aromatic rings instead of the saturated ones, the similarity score drops (see compounds C and D of Fig. 6). So, it can be observed that the most similar molecule to the Perindopril according to the fingerprint similarity score is not the solution that includes the Perindopril. As this score is an average over all atom environments, the addition of any new environment absent from the Perindopril has a huge impact. Therefore, EvoMol finds the smallest possible aromatic ring in order not to degrade the similarity score too much and increase the number of alkyl carbons to compensate.

It appears that the fine definition of a property profile (MPO) is difficult. Furthermore, the choice of fingerprints and Tanimoto distances does not correspond to a chemical neighbourhood as defined by the actions on the molecular graph. Even more problematic, it does not seem possible to us to define chemically interpretable actions to make our neighbourhood correspond to the distances between fingerprints. Therefore, it is not a good objective function to guide our method towards improving solutions.

With atom centred actions, EvoMol is a quite unconstrained molecule generator. The chemical soundness of the solutions has to be verified. In GuacaMol, a compound quality measurement is provided. 77% of the GuacaMol initial dataset pass this test. In this test evolutionary algorithms do not perform as well as RNN methods. For our method (all actions model), 34% pass the quality check, quite similarly to comparable methods [36]. However, this score covers a wide variety of situations depending on the benchmark. For instance, most isomers of $\hbox {C}_{11}\hbox {H}_{24}$ pass the quality filter (more than 98%). In the case of Perindopril, where the aim is to obtain a similar solution, 84% of the proposed solutions are considered as valid. On the contrary, when the aim is to find a molecule chemically opposed to Sitagliptin, EvoMol only proposes molecules that do not pass (100%) this filter based on the presence of fragments prohibited in medicinal chemistry. We performed a detailed quality study for all our models and all benchmarks, available in Additional file 3. It shows that using a dataset of molecules as initial population and the set of secondary actions generally improves the quality of generated solutions.

Since EvoMol is designed to be flexible, we can implement an obligation to pass this filter in the generation process thus ensuring 100% validity of the solutions across all the research path. With this very conservative constraint, the mean score obtained on ten executions is 0.897. Among twenty, this task is the only one evaluating the ability of generators to propose molecules far from what is already known. EvoMol is able to handle both the multi-objective optimisation problem and validity constraints to deliver high performance. For the Osimertinib objective, the ratio of solution passing the quality measurement test range from 0% to 70%. Applying the filtering during the generation process allow us to reach a mean score of 0.959 with 100% validity and therefore without impacting performance. We have applied this methodology for a full run on all GuacaMol benchmarks. This constraint, the obligation to pass this quality filter is, as expected, not enough to ensure a chemical feasibility. Top scores for the MPO with and without the quality filter are depicted in Additional file 4. The evaluation of the chemical feasibility is still far from perfect. Thus, the evaluation function of evolutionary models suffers from this lack. For drug design, the main solutions proposed to avoid this issue are fragment-based, reaction-based or deep generative models [49]. For case 3 we will look at how introducing bias, by means of the CLscore in the objective function, can improve the quality of the generated results.

**Fig. 7:**
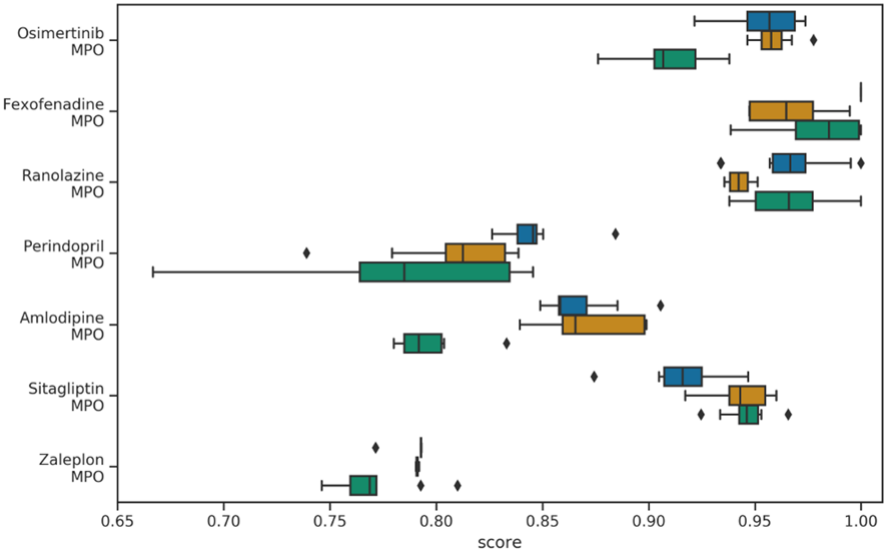
Boxplot of selected GuacaMol benckmarks scores obtained on 10 executions of each experiment with EvoMol. Experiments using all actions, using only primary actions and starting from methane are represented in blue, orange and green respectively

Thanks to the different runs of our models, it is possible for once to appreciate the variability of these objectives as shown in the following Fig. 7 (the full Figure showing the box plots for all the benchmarks is included in the Additional file 5). In this Fig. 7, for each objective, we can see the dispersion over 10 runs with all the actions, only the primary actions and all the actions but starting from methane. It can be seen that in the latter case, the model is clearly at a disadvantage. The dispersion is very large, but it is likely that with more steps some executions would have reached their objectives and others would have remained blocked. One can also note the generally positive effect of secondary actions that worked with half as many steps. Inspired by Henault et al. in their exploration of the chemical space with a genetic algorithm, we can look more precisely at our efficiency in the rediscovery tasks [46]. In Table 4 we note that the secondary actions have a huge impact on the number of calls to the evaluation function in order to find the target. For the Celecoxib they are even necessary. GraphGA, as a genetic algorithm, contains a pivotal crossover operator. It allows to perform big leaps in the chemical space, while conserving valuable fragments. EvoMol, with its set of seven actions and without crossover, seems to provide performances of the same order of magnitude.

**Table 4:** Study of the impact of the secondary actions on the efficiency of EvoMol on the Guacamol rediscovery benchmarks

Rediscovery benchmark	Primary actions		All actions	
	Success rate	Eval. ($\times 10^6$)	Success rate	Eval. ($\times 10^6$)
Celecoxib	0.0	–	0.9	0.37
Troglitazone	0.7	2.32	1.0	0.50
Thiotixene	0.4	11.1	0.4	0.81

The success rate is evaluated on 10 executions, considering a success when the target molecule is exactly found. The reported numbers of evaluations (in millions) is the mean of the successful executions

In the end, with our different executions, we can observe a variability between the different runs easily of the order of 0.05 for the most complex metrics. We can therefore estimate that a difference on the total score of the order of 0.2 is not significant. Thus it is likely that EvoMol, GraphGA, CREM and MSO are in fact comparable in terms of pure optimisation performance on this set of benchmarks.

### Case 3: HOMO/LUMO optimisation

The last case consists in optimising an electronic property. The frontier molecular orbital levels are key points for any reactivity problems, electroactive molecules and electronic or photonic organic materials. So, we choose to focus on the HOMO and LUMO energies. The energy levels are obtained with a geometric optimisation calculation in DFT.

**Fig. 8:**
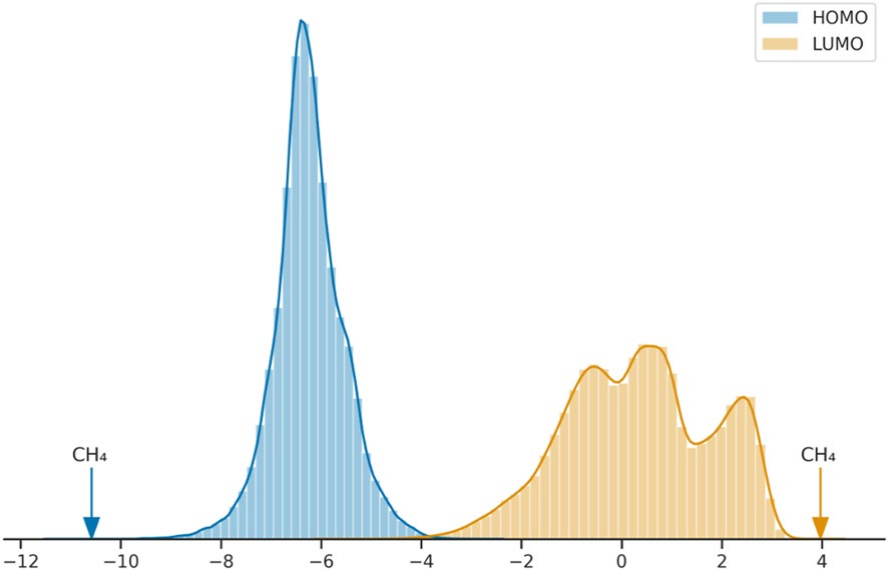
Frontier Molecular Orbitals energies distribution in the union of QM9 and PC9 datasets. Values of methane are indicated with arrows

In a previous article we studied the QM9 and PC9 datasets that together encompass more than 200k different molecular calculations with up to 9 heavy atoms of C, N, O and F types [31]. Initially computed with different methods, we relaunched them using our BOINC collaborative computing project, called QuChemPedIA@home, in order to have a homogeneous and clean dataset. Figure 8 presents the distributions of these MO energies in the union of QM9 and PC9.

One could hope for a fast evaluation of quantum mechanics properties thanks to machine learning predictions to limit the cost of computation. However, we have demonstrated that the currently available datasets of molecular quantum chemistry results, like QM9 and PC9, are not diverse enough to train a general predictor [31]. It is clear that solving this issue in the future would significantly accelerate the generation of molecules with such objectives. Meanwhile, DFT calculations are mandatory for the exploration process in this problem.

The data for methane are highlighted in Fig. 8 to show that this molecule has one of the lowest HOMO energies and one of the highest LUMO energies. It is a very poor candidate choice when the specification is often an ability to give electrons (high energy HOMO) or an ability to yield electrons (low energy LUMO). We have therefore tested the ability of EvoMol to optimise the HOMO energy (to the highest) and LUMO energy (to the lowest) starting from only a methane molecule. To make the optimisation tractable, we set the population size to 20 and the number of replaced individuals at each step to 2. The solutions can have up to 9 heavy atoms of the same kind as QM9 and PC9.

**Fig. 9:**
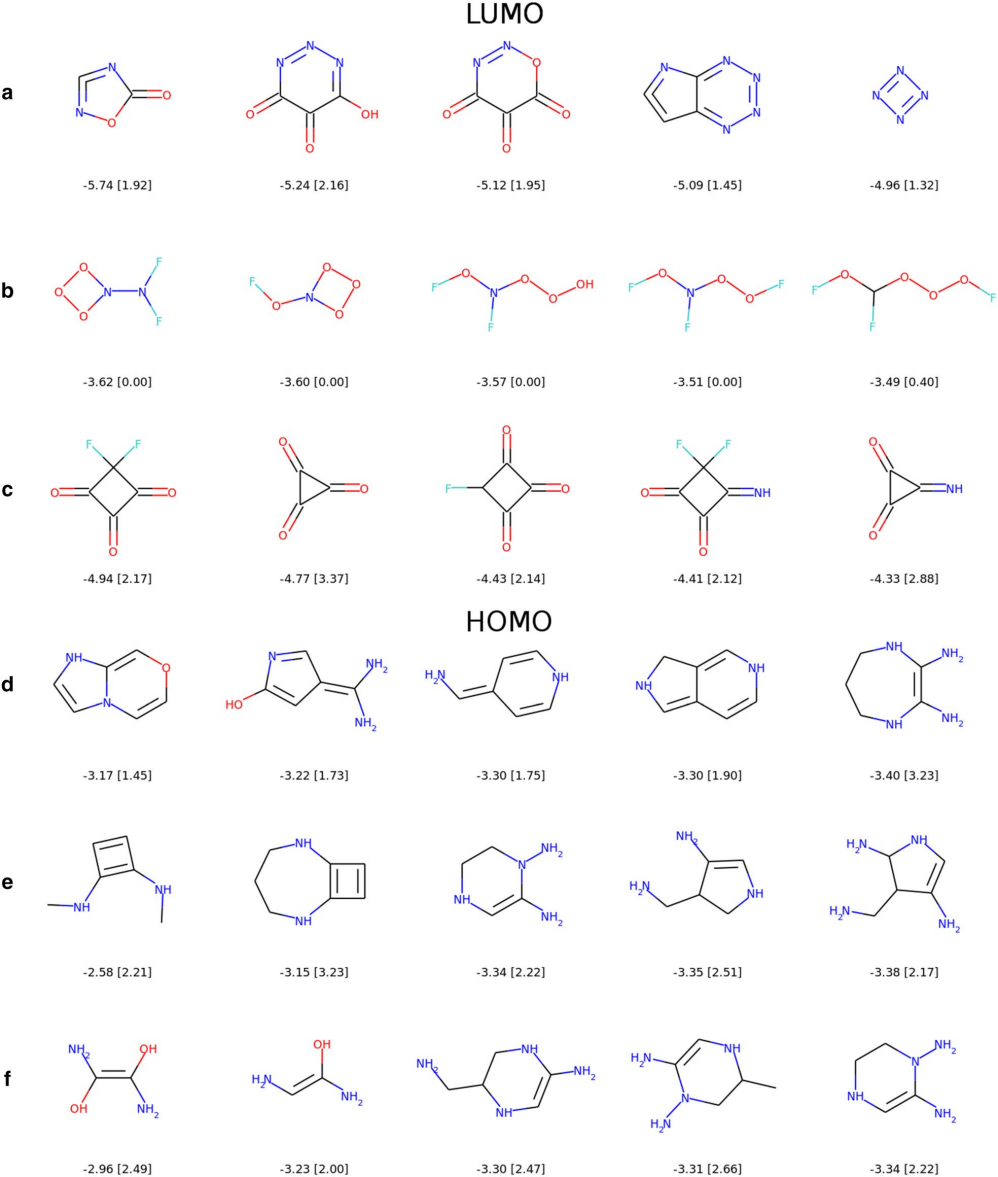
Best five solutions for LUMO (up) and HOMO (down). For both properties, the first line corresponds to best values in QM9$\cup$PC9 after removing zwitterions and radicals (**a**, **d**). The second line corresponds to the EvoMol experiment without any synthesizability constraint (**b**, **e**), and the last line corresponds to the joint optimisation with the CLscore constraint (**c**, **f**). The values of the properties (HOMO or LUMO [CLscore]) are reported under the molecules

Figure 9 presents the best five solutions of the QM9 and PC9 datasets (without radicals and zwitterions) and those obtained with EvoMol. For the LUMO energies, QM9 and PC9 lowest values correspond to highly nitrogenous cycles and carbonyl groups. It is worth pointing out here that the compounds 1 and 3 correspond probably to very strained structures due to cumulated double bonds in a 5-member ring, and that the tetranitrogen is also very unstable.

Our first attempt with EvoMol (line B of Fig. 9) has found that discarding all carbons makes it possible to reach low energy levels. Some kind of nitro compounds are proposed and fill all the population of 20 individuals. It cannot leave and improve from this chemical territory with only one mutation. In view of the proposed solutions, we have calculated their corresponding CLscore values as an estimation of their likelihood. They are almost all null because this chemistry does not exist in ChEMBL. Therefore, in order not to get lost in too exotic territories, we have modified our objective function to include a CLscore based constraint. To get a smooth and continuous objective function [50], we use the product of two sigmoid functions, one for the electronic property and a one for the CLscore (see Eq. 1). The CLscore sigmoid is centred around a value of 1.5. It blocks the appearance of compounds with values below 1 and penalises the solutions with a CLscore between 1 and 2. The sigmoid functions for HOMO and LUMO are set so that they are roughly centred in the middle of the distributions (Fig. 8) and reach their maximum values for the best known solutions.

1 $$\begin{aligned} \begin{aligned} f_{\mathrm {CLscore}}(x)&= \frac{1}{1 + e^{10(-x + 1.5)}}\\ f_{\mathrm {LUMO}}(x)&= \frac{1}{1 + e^x} \\ f_{\mathrm {HOMO}}(x)&= \frac{1}{1 + e^{-x - 7}} \end{aligned} \end{aligned}$$

The solutions corresponding to this multi-objective function are drawn in line C of Fig. 9. We observe an intensification around the carbonyl group and the fluorine (known to serve as acceptor functions). Even if the CLscore of the cyclopropanetrione is above 3, it does not mean that it is a stable compound. It was only detected during Mass Spectrometry experiments but seems to be a promising target for energy storage application. Thus this simple LUMO energy test led to the rediscovery of a real target sought in molecular materials [51].

For the HOMO, the chemistry of the amine function was quickly found and intensified (lines E and F). The CLscore filter allows for greater diversity but has less impact on the solutions. Indeed in QM9 and PC9 (line D), the compounds with the highest HOMO energies are also amine derivatives (more often aromatic). Ultimately, we can notice that our method allows us to get close to the best energies in HOMO and LUMO starting from a worst case scenario.

**Fig. 10:**
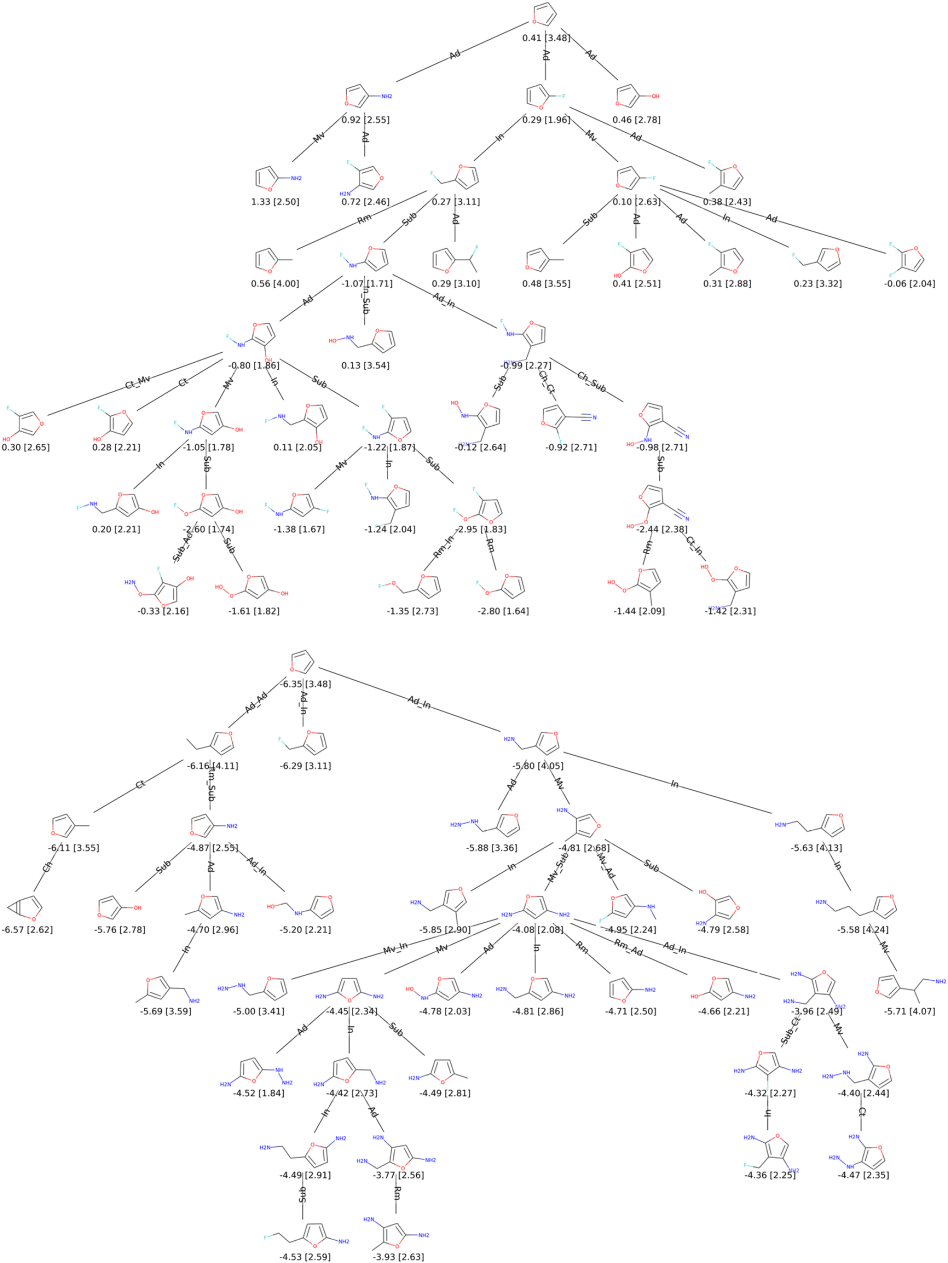
Exploration trees for the minimisation of LUMO (top), and the maximisation of HOMO (bottom) energies starting from a fixed furane core. Energy values (in eV) and [CLscores] are reported under the solutions. The actions used for the transition between two molecules are reported on the edges with the following legend. Ad: *append atom*, Rm: *remove atom*, Ch: *change bond*, Sub: *substitute atom type*, In: *insert carbon*, Ct: *cut atom* and Mv: *move group*

To be more realistic, we end up with an experiment that adds a structural constraint. We impose a furane core which is an aromatic ring resembling the conjugated systems used in organic materials. The research space is therefore more limited with 4 more heavy atoms to generate. Both HOMO and LUMO are also optimised with the CLscore sigmoid. Thanks to our approach with mutations acting near the atom level, we can propose an interesting visualisation of the exploration process for this experiment. In Fig. 10, all improvers are represented as nodes in a tree whose edges are labelled with actions. We set in this case the population size to 10 to allow for a reasonably sized visualisation.

In the upper part of the figure, we can see that for the HOMO EvoMol tries alkyl, fluorine, alcohol and amine substitutions. We also notice that the operation of moving a substituent allows it to probe the interest of the different positions and their combinations. From there, an intensification takes place around the poly amine improvers. In the lower part, for the LUMO objective, the amine and alcohol are also probed but rapidly discarded. The first effective branch is associated with fluorine substitution, which then becomes more complex. It is worth noting the appearance of cyano and especially peroxo and oxygen fluoride functions which allow very good values for realistic solutions.

These exploration trees mimic the way of working of a human chemist who, around efficient fragments, walks through the chemical neighbourhood in elementary steps. By this Fig. 10, we can underline the superior interpretability of this atom-centred approach compared to fingerprints for example. Moreover, we show that our well-defined objective function is able to properly guide the exploration process on this challenging task.

## Conclusions

In this article, we present EvoMol, an efficient evolutionary algorithm for molecular generation. By design, it generates only valid molecular graphs and unique solutions. EvoMol can be used with a maximum population size of one molecule. In optimisation, this corresponds to a very naive approach called the hill-climbing algorithm. This simple climber finds a direct pathway to very good solutions for classical objective functions (QED, penalised logP). Therefore, it showcases the limitations of such objectives as benchmarks. EvoMol can also generate large datasets of high scoring solutions, by intensifying thoroughly on promising areas of the chemical space.

We show in this article that EvoMol is highly configurable and provides state of the art performances for both classical drug design (QED, penalised logP, SAscore, GuacaMol benchmarks) and molecular material problems. Contrary to many methods, EvoMol does not require prior knowledge extracted from a dataset to obtain high scoring solutions. It can therefore tackle very different problems and explore different chemical subspaces depending on the objective. We demonstrate that in EvoMol, bias can be strictly controlled. Prior knowledge can be introduced easily through the objective function, filters or the starting dataset.

The mutation operator is composed of 7 general atom centred actions that define an interpretable neighbourhood on the molecular graph. As the exploration process only depends on this operator, it becomes thoroughly traceable and allows for chemically meaningful visualisations. This interpretability will be useful for further fine-tuning of the exploration process. The optimum found with EvoMol highlighted several limitations on the commonly used objective functions. For instance, we have found that using fingerprints can lead to counter-intuitive distances between molecules.

EvoMol shows good performances in optimisation but for a practical application this is not satisfactory, since the quality of the compounds is an essential criterion. Evaluating the quality of a molecule is for the moment either very subjective or an imperfect measurement and it is an active research field. This is also the weakness of evolutionary methods in general [52]. For drug design, the main solutions proposed are fragment-based, reaction-based or deep generative models. For domains were data are scarce, we think that a flexible and unbiaised generator can be a good starting point in order to control the needed bias for quality control. Concerning this aspect, EvoMol offers several possibilities. Providing an objective function such as SAscore or CLscore and combining it with others is the most obvious solution. This solution alone is rarely good enough. Fragment-based approaches are one way to address this problem. Using EvoMol filters, it is easy to implement either a whitelist or a blacklist of fragments. With a whitelist, EvoMol would become a full fragment-based evolutionary algorithm.

Thanks to the tree visualisation, we could observe how EvoMol builds and improves upon the furane core like an experimental chemist playing very efficiently with substitutions and positions and then intensifying on promising candidates. Since the synthesizability prediction is still a complex issue, we believe that this related solution tree could be a useful tool to discuss chemistry. It brings a definite added value compared to a simple portfolio because it explains the trial-and-error of the exploration and the proximity of the proposed solutions.

In the future, we believe our method would benefit from a diversity mechanism. It would help to provide high scoring individuals in various areas of the chemical space. It should be configurable in order to choose a compromise between exploration and intensification depending on the objective. We also consider that EvoMol could profit from reinforcement learning to select actions depending on the context. This should limit the number of calls to the evaluation functions, especially in the case of a costly objective function.

## Supplementary information

**Additional file 1.** List of the 457,139 SMILES of the ChEMBL-like dataset with their corresponding CLscore.

**Additional file 2.** Archive of the GuacaMol benchmarks experiments.

**Additional file 3.** Proportion of generated solutions passing GuacaMol quality benchmark.

**Additional file 4.** Representation of the best solutions found on GuacaMol MPO benchmarks.

**Additional file 5.** Boxplot of all 20 goal-directed GuacaMol benckmarks scores obtained on 10 executions of each experiment with EvoMol. Experiments using all actions, using only primary actions and starting from methane are represented in blue, orange and green respectively.

## Data Availability

The EvoMol code source is available in the following github repository : https://github.com/jules-leguy/EvoMol
