# Supplementary material for: EvoMol: a flexible and interpretable evolutionary algorithm for unbiased de novo molecular generation
Source: J Cheminform. 2020 Sep 16;12:55. doi: 10.1186/s13321-020-00458-z (PMC7494000; doi:10.1186/s13321-020-00458-z)
Supplement: Supplementary file 3 — Additional file 3. Proportion of generated solutions passing GuacaMol quality benchmark. [file 13321_2020_458_MOESM3_ESM.pdf]

GuacaMol benchmark	Primary actions	All actions	From methane
Celecoxib rediscovery	90	100	90
Troglitazone rediscovery	0	0	0
Thiotixene rediscovery	70	60	30
Aripiprazole similarity	19	62	32
Albuterol similarity	17	35	13
Mestranol similarity	47	67	33
$C_{11}H_{24}$	99	99	99
$C_9H_{10}N_2O_2PF_2Cl$	9	11	4
Median molecules 1	72	90	90
Median molecules 2	24	39	13
Osimertinib MPO	9	33	25
Fexonadine MPO	0	7	1
Ranolazine MPO	0	0	0
Perindopril MPO	65	84	47
Amlodipine MPO	20	3	32
Sitagliptin MPO	8	0	4
Zaleplon MPO	31	31	29
Valsartan SMARTS	0	10	21
deco hop	4	2	0
scaffold hop	2	1	0
total	26	34	27

Proportion of solutions passing the GuacaMol quality test (%).
