# Supplementary material for: EvoMol: a flexible and interpretable evolutionary algorithm for unbiased de novo molecular generation
Source: J Cheminform. 2020 Sep 16;12:55. doi: 10.1186/s13321-020-00458-z (PMC7494000; doi:10.1186/s13321-020-00458-z)
Supplement: Supplementary file 4 — Additional file 4. Representation of the best solutions found on GuacaMol MPO benchmarks. [file 13321_2020_458_MOESM4_ESM.pdf]

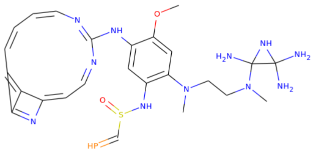

Osimertinib MPO [0.979, 0]

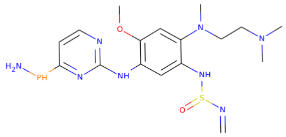

Osimertinib MPO [0.946, 1]

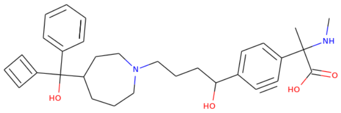

Fexofenadine MPO [1.000, 0]

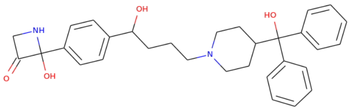

Fexofenadine MPO [1.000, 1]

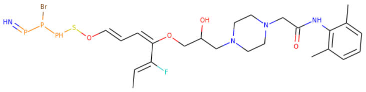

Ranolazine MPO [1.000, 0]

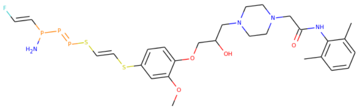

Ranolazine MPO [0.987, 1]

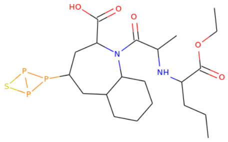

Perindopril MPO [0.887, 1]

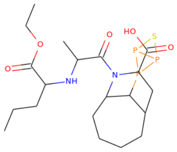

Perindopril MPO [0.891, 1]

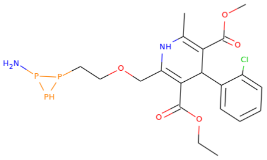

Amlodipine MPO [0.906, 1]

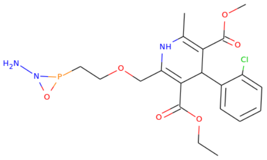

Amlodipine MPO [0.906, 1]

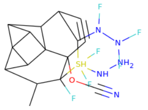

Sitagliptin MPO [0.966, 0]

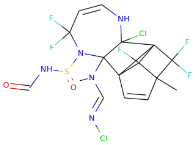

Sitagliptin MPO [0.878, 1]

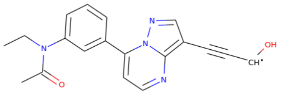

Zaleplon MPO [0.835, 1]

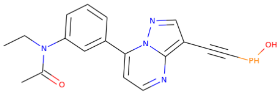

Zaleplon MPO [0.794, 1]

Best scoring solutions obtained for GuacaMol MPO benchmarks optimization using EvoMol. Benchmark name : [Benchmark score, quality filter value]. Left : best solutions found across all runs (primary actions, all actions, from methane). Right : best solutions found using the quality filter constraint.
