# Supplementary figures and images for: EvoMol: a flexible and interpretable evolutionary algorithm for unbiased de novo molecular generation
Source: J Cheminform. 2020 Sep 16;12:55. doi: 10.1186/s13321-020-00458-z (PMC7494000; doi:10.1186/s13321-020-00458-z)

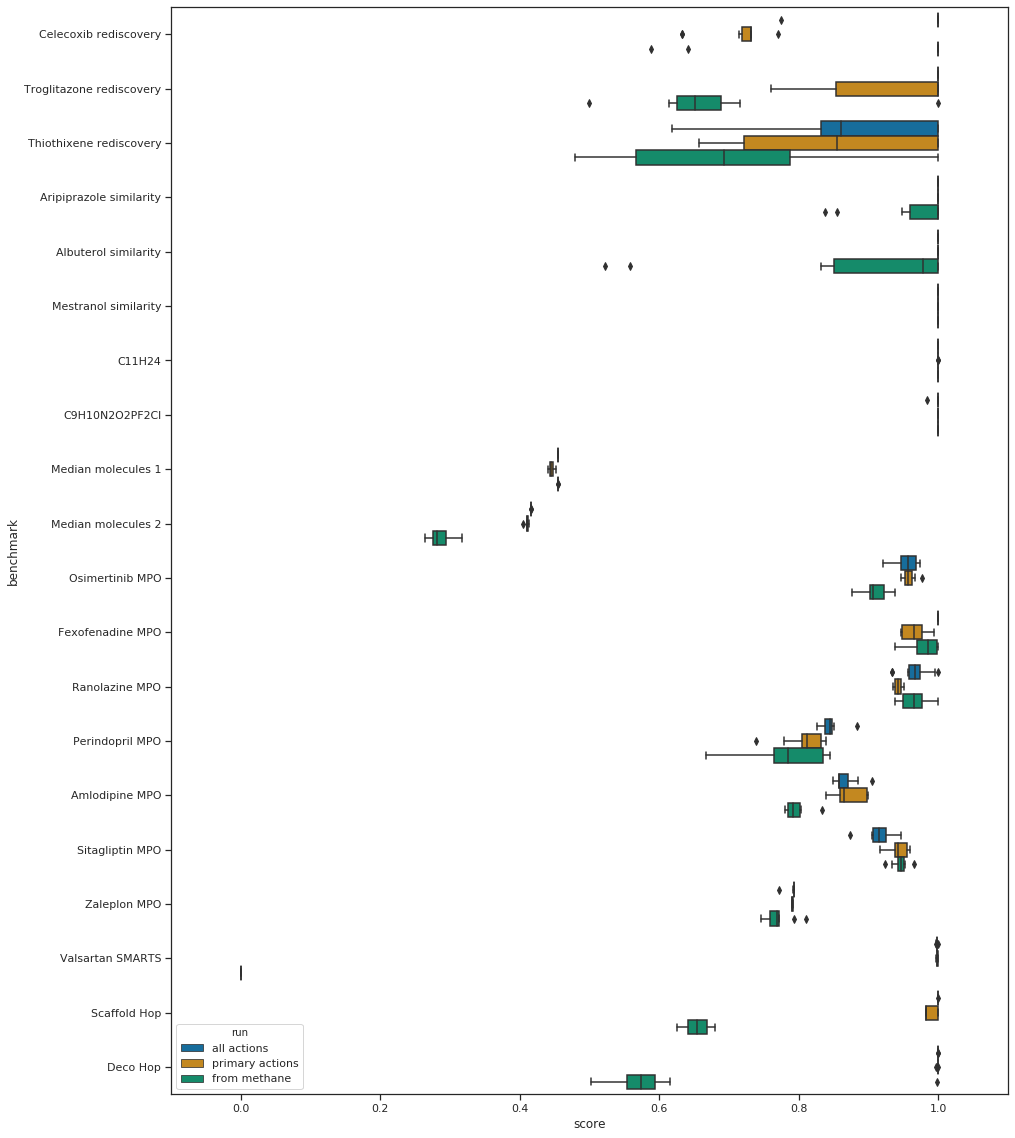

Supplement: Supplementary file 5 — Additional file 5. Boxplot of all 20 goal-directed GuacaMol benckmarks scores obtained on 10 executions of each experiment with EvoMol. Experiments using all actions, using only primary actions and starting from methane are represented in blue, orange and green respectively. [file 13321_2020_458_MOESM5_ESM.png]
